# Digital Marketing to Promote Healthy Weight Gain Among Pregnant Women in Alberta: An Implementation Study

**DOI:** 10.2196/11534

**Published:** 2019-02-01

**Authors:** Jocelyn E Graham, Jana L Moore, Rhonda C Bell, Terri Miller

**Affiliations:** 1 Reproductive Health Population, Public and Indigenous Health Alberta Health Services Calgary, AB Canada; 2 Department of Agricultural, Food & Nutritional Science Faculty of Agricultural, Life & Environmental Sciences University of Alberta Edmonton, AB Canada

**Keywords:** internet, maternal health, mobile phone, pregnant women, search engine, social media

## Abstract

**Background:**

As the use of digital media for health promotion has become increasingly common, descriptive studies exploring current and innovative marketing strategies can enhance the understanding of effective strategies and best practices.

**Objective:**

This study aims to describe the implementation of a provincial digital media campaign using complementary advertising platforms to promote healthy pregnancy weight gain messages and direct a Web audience to a credible website.

**Methods:**

The digital media campaign occurred in 3 phases, each for 8 weeks, and consisted of search engine marketing using Google AdWords and social media advertising through Facebook. All advertising materials directed users to evidence-based pregnancy-related weight gain content on the Healthy Parents, Healthy Children website.

**Results:**

Google Ads received a total of 43,449 impressions, 2522 clicks, and an average click-through rate (CTR) of 5.80%. Of people who clicked on a Google ad, 78.9% (1989/2522) completed an action on the website. Across all Facebook advertisements, there were 772,263 impressions, 14,482 clicks, and an average CTR of 1.88%. The highest-performing advertisement was an image of a group of diverse pregnant women with the headline “Pregnancy weight is not the same for every woman.”

**Conclusions:**

This study supports the use of digital marketing as an important avenue for delivering health messages and directing Web users to credible sources of information. The opportunity to reach large, yet targeted audiences, along with the ability to monitor and evaluate metrics to optimize activities throughout a campaign is a powerful advantage over traditional marketing tactics. Health organizations can use the results and insights of this study to help inform the design and implementation of similar Web-based activities.

## Introduction

Studies have consistently found that the majority of Canadian women do not meet the Health Canada [1] gestational weight gain (GWG) recommendations [2,3]. Exceeding these recommendations is associated with adverse outcomes, such as pre-eclampsia, cesarean delivery, and infants born large for gestational age [4], with an increased risk of childhood obesity [5]. Weight gain below the recommendations can increase risks of preterm birth and infants who are small for their gestational age [6]. Reported barriers to meeting GWG recommendations include misperceptions, lack of advice, and poor knowledge with regard to personal body mass index, GWG recommendations, and the impact of GWG on health outcomes [7-10].

While the prenatal visit offers an opportunity to provide pregnancy information and support, many women feel that discussions with health professionals are insufficient and use Web-based resources to compensate [11,12]. Women commonly access the internet during pregnancy [13,14] and perceive Web-based health information as reliable and useful, while appreciating features such as anonymity, simplicity, and unrestricted access at any time [15,16]. In a study by Larsson [17], 84% of women reported using the internet to obtain pregnancy-related information, particularly in early pregnancy, and 97% of participants in a study by Lagan et al [12] reported using search engines to find pregnancy webpages. In addition, women use social media, including Facebook, during pregnancy to share their experiences and seek advice, with the majority accessing these platforms, at least, once a day [11,15].

The internet, therefore, provides an opportunity to increase women’s awareness and knowledge about healthy GWG and connect women with credible resources, as other Web-based information may not be consistent and evidence-based. For health organizations, advantages of using digital platforms for these purposes include low cost, accessible information, interactivity with users, and the ability to deliver tailored messages with a wide reach to specific audiences [18,19]. Because of these advantages, it has become increasingly common for health organizations to create and maintain social media accounts. Organizations can share health information through these accounts in 2 main ways—unpaid (organic) and paid (advertising). As social media platforms, like Facebook, continue to decrease the amount and frequency of content from organizations that users see organically [20], paid advertising is becoming increasingly important. Descriptive studies about the feasibility and steps for implementing successful Web-based campaigns are needed to support a better understanding of innovative strategies that engage the public in health topics like GWG [18,19,21].

Informed by recent Alberta-based maternal health research [3,10,22], a provincial marketing campaign using digital platforms was developed to promote awareness of healthy GWG among women. This paper aims to describe the development and implementation of the digital campaign, which used search engine marketing using Google AdWords and social media advertising through Facebook. This project was a collaboration between Alberta Health Services (AHS), which is the health authority for the Canadian province of Alberta, and the University of Alberta’s ENRICH Research Program.

## Methods

### Campaign Objectives

The GWG campaign was embedded within *Healthy Parents, Healthy Children* (HPHC), a program created by AHS. This program provides evidence-based information and best practice advice to expectant parents and parents of children up to 6 years of age through printed books, a website [23], and social media activity. Pregnancy information on the website covers topics such as nutrition, exercise, distribution of weight, risks of gaining too much or too little weight, and weight gain guidelines. A Web-based calculator is also available for pregnant women to find their prepregnancy body mass index and recommended weight gain range as well as to track their current weight in relation to their recommendation [24]. All digital advertising campaign materials directed users to this GWG content on the HPHC website.

The campaign objectives were to increase awareness about the importance of healthy GWG among women in Alberta and direct women to the HPHC website. Google AdWords and Facebook Ads were chosen to achieve these objectives as Google is the most popular search engine [25] and Facebook is the most used social media network, with the greatest proportion of users being women aged 18-29 years [26]. In addition, the combination of these platforms reaches 2 audiences—those searching for information (Google AdWords) and those who may not be actively searching (Facebook). A health marketing framework that “involves creating, communicating, and delivering health information and interventions using customer-centered and science-based strategies to protect and promote the health of diverse populations” [27] was used to guide the campaign.

### Google AdWords

Google AdWords is a paid service that displays ad text and a website link above, beside, or below a list of Google search results when a user performs a search using keywords that match those selected by the advertiser. In the list of search results, these paid ads are labeled “Ad” to differentiate them from organic search results.

### Facebook Ads

On Facebook, ads are paid messages from organizations or businesses displayed to members of a predefined audience. Paid ads look similar to unpaid Facebook content and can appear throughout the social network. Advertisers can set budget, articulate advertising objectives, and define a target audience, all of which determine where, how often, and to whom the ad is shown. For this campaign, the project team developed the ads using Facebook Ads Manager, Facebook’s advertising management platform.

### Campaign Design

The campaign occurred in 3 distinct phases of 8 weeks each between January 2 and December 3, 2017, with a total budget of Can $7034.44. This design was used to decrease the risk of message fatigue and overexposing the audience, which would have been more likely had this been carried out as a single 24-week campaign. To ensure equitable distribution across the entire province, the budget was allocated to 3 markets—65% (Can $4572.39/Can $7034.44) to 2 major cities in Alberta, 15% (Can $1055.17/Can $7034.44) to smaller cities throughout the province, and 20% (Can $1406.88/Can $7034.44) to rural Alberta. A local vendor was contracted to establish the advertising platforms and manage the first phase of the campaign. The remaining phases of the campaign were maintained by internal staff who attended local and Web-based digital marketing training sessions to gain knowledge and expertise. At the end of each campaign phase, staff reviewed the campaign results and adapted the strategy for the next phase.

**Figure 1:**
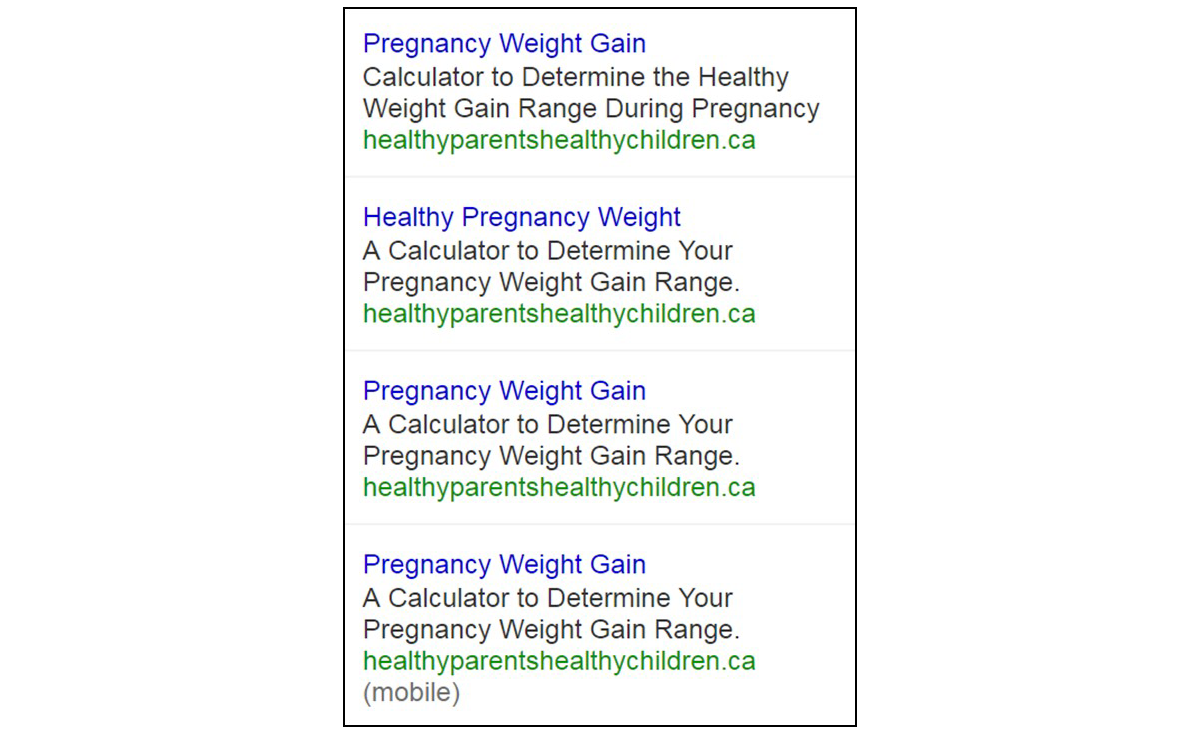
Sample design of Google AdWords advertisements promoted during the campaign.

Overall, 3 Google AdWords ads were developed to be used across all phases (Figure 1) and set to appear to users in Alberta searching for pregnancy weight information. One of the ads was duplicated and served only to mobile devices. When a user clicked on the ad, they were brought to the GWG calculator on the HPHC website. Google AdWords uses a bidding system to determine which ads are displayed and the cost to the advertiser. For this digital campaign, the budget was Can $10 per day, and we used automatic cost per click (CPC) bidding, meaning we only paid when the ad was clicked.

For Facebook ads, the audience was defined as female, aged 18-44 years, with interests in pregnancy. Ads were selected to show on all devices (mobile, tablet, and desktop) and in Facebook newsfeeds, instant papers, and in the right column of Facebook’s desktop platform. A flexible daily budget of Can $26 was set to have ads run continuously. Like Google AdWords, Facebook uses a bidding system to determine which ads to display. For the digital campaign, Facebook performed automatic bidding based on campaign objectives and audience to achieve the highest number of clicks on the ad at the lowest cost. Allowing for adjustments in daily spending through the automatic bidding process helped to take advantage of opportunities in the ad auction marketplace to optimize results.

**Figure 2:**
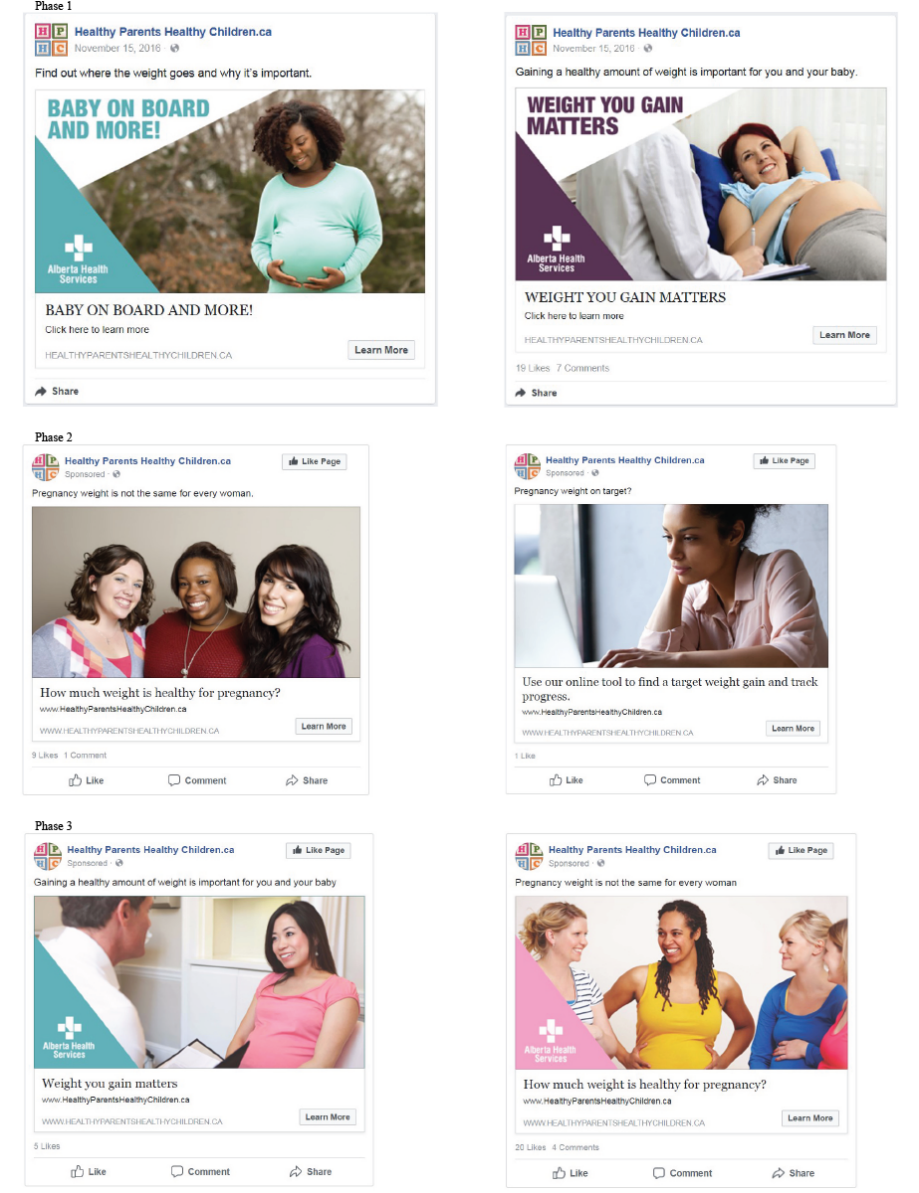
A screenshot of the Facebook advertisements for each campaign phase. (Source: photos purchased from iStock.com and Shutterstock.com).

Unlike Google AdWords, Facebook ads offer the opportunity for longer messaging, images, and branding. Two new HPHC-branded Facebook ads were developed by staff prior to each phase of the campaign using key messages about healthy pregnancy weight, stock photos, and a link to a relevant HPHC webpage (Figure 2). Ads adhered to Facebook guidelines; however, adjustments to the wording of the ad copy were required when Facebook did not approve ads because of an unexpected application of their prohibited content guidelines that restricted the use of the phrase “weight gain” and the option to ask personal questions (eg, How much weight gain is healthy for you during pregnancy?).

During the first phase, ads incorporated color blocks using HPHC branding colors and text onto the image. For the second phase, women in the images were not visibly pregnant and the color blocks and text were removed. Ads for the third phase incorporated design and content elements of the top performing ads from the previous phases; these elements included an image of a woman talking with a health care provider, an image of diverse women to promote the individuality of weight gain, displaying the AHS logo, and the use of HPHC branding colors.

Facebook users can react and leave comments on paid ads; hence, throughout the campaign, staff monitored the ads for comments and provided timely responses, redirecting users to additional HPHC webpages as applicable. While privacy and profanity issues were not a challenge during the campaign, staff had prepared to hide comments that were inappropriate or revealed personal medical information, and connect with users through private messaging as needed.

### Data Analysis

Both Google and Facebook provide extensive data for paid ads delivered using their platforms. Ad performance data were collected from the HPHC Google AdWords account and Facebook Ads Manager. Definitions of key metrics can differ across digital platforms. For the results and discussion that follows, we will use the following terms and definitions:

- Impressions: the number of times an ad was displayed to a user
- Engagement: interaction with an ad, including Facebook user reactions, sharing, and comments, as well as ad clicks
- CPC: price an advertiser pays for each click on the ad
- Click-through rate (CTR): the number of clicks on the ad per the number of impressions
- Conversions: actions that a user completes after clicking on an ad (completing the Web-based weight gain calculator, clicking to download the results of the Web-based weight calculator, clicking on a social media share button). These data could be collected because of the existing codes that had been added to the HPHC website by the website developer.

It was predicted that Facebook ad engagement would be low because weight can be a personal and sensitive topic, and many women in early pregnancy delay public announcement. Any reaction, comment, or share of the ads could be seen by the women’s Web-based social network, which may deter users from engaging in these ways. For this campaign, the CTR was a better indicator of overall success and user interest in the ad message.

## Results

### Google AdWords

Ads displayed on Google received a total of 43,449 impressions throughout the campaign. The average position of a campaign ad in the search results list was 1.3, meaning the ad was often displayed first or second when an associated keyword was searched. A total of Can $1913.72 was spent on ads with an average CPC of Can $0.76, which is the average amount charged for a click on an ad.

The Google ads were clicked 2522 times, with an average CTR of 5.80%. The most popular search term that led to an ad click was “calculate weight gain during pregnancy” with 137 clicks (Table 1). The search term “pregnancy weight gain” had the highest CTR at 24.07% (Table 2). There were 1989 conversions as a direct result of the ads, which represents a 78.9% (1989/2522) conversion rate by users who clicked on an ad.

### Facebook Ads

During the campaign, Facebook ads received 772,263 impressions, and the average number of times an ad was displayed to the same individual ranged from 2.53 to 3.28. A total of Can $5067 was spent on Facebook ads with an average CPC of Can $0.35. A greater amount was spent on Facebook ads because of the platform’s required minimum daily budget for an ad set. Table 3 shows results for Facebook ads during each phase. The overall average CTR was 1.88% (range 0.32%-2.56%), and the most clicks on any one ad were 4741. In comparison, the highest number of clicks for organic, unpaid Facebook posts on the HPHC account during 2017 that included an image and a link to a healthy pregnancy weight gain content page was 95. The highest-performing ad, as determined by the CTR, occurred in the third phase and included the AHS logo, an image of diverse women, and the headline “Pregnancy weight is not the same for every woman” (Figure 2).

Facebook ads were clicked a total of 14,482 times, for an overall CTR of 1.88%. Ads received 43 comments, 28 shares, and 247 reactions. While a rigorous content analysis was not performed, the majority of ad comments were neutral, with many users sharing about their personal weight gain experience. Several comments provided an opportunity to enhance knowledge. For example, when a woman commented on weight gain for carrying twins, our reply encouraged them to talk with her health care provider, and we shared a link to the HPHC webpage containing information about healthy weight gain for a twin pregnancy.

**Table 1:** Top 5 performing keywords on Google AdWords by ad clicks.

Keyword	Clicks, n (%)	Click-through rate, %	Cost per click, Can $	Cost, Can $
Calculate weight gain during pregnancy	137 (5.43)	10.65	0.65	88.91
Pregnancy weight gain chart calculator	109 (4.32)	17.90	0.54	58.39
Weight gain during pregnancy chart	103 (4.08)	12.06	0.58	59.96
Pregnant weight gain chart calculator	90 (3.57)	13.72	0.55	49.72
Calculate weight gain during pregnancy	89 (3.53)	8.52	0.64	56.65

**Table 2:** Top 5 performing keywords on Google AdWords by click-through rate.

Keyword	Clicks, n (%)	Click-through rate, %	Cost per click, Can $	Cost, Can $
Pregnancy weight gain	13 (0.52)	24.07	0.39	5.10
Healthy pregnancy weight gain chart	4 (0.16)	20.00	0.65	2.61
Weight gain during pregnancy month by month	6 (0.24)	20.00	0.75	4.50
Pregnancy weight gain chart calculator	109 (4.32)	17.90	0.54	58.39
Healthy pregnancy weight gain chart	6 (0.24)	17.65	0.50	03.01

**Table 3:** Facebook Ads results during each phase.

Phase and ad headline		Impressions, n (%)	Clicks, n	Click-through rate, %	Cost per click, Can $	Comments, n	Shares, n	Reactions, n
**Phase 1**								
	Find out where the weight goes and why it’s important	27,747 (3.59)	485	1.75	0.41	0	0	34
	Gaining a healthy amount of weight is important for you and your baby	197,921 (25.63)	4273	2.16	0.37	22	8	89
**Phase 2**								
	Pregnancy weight is not the same for every woman	295,762 (38.30)	4741	1.60	0.34	11	6	47
	Pregnancy weight on target?	60,522 (7.84)	196	0.32	0.17	0	2	4
**Phase 3**								
	Pregnancy weight is not the same for every woman	176,288 (22.83)	4521	2.56	0.33	10	12	62
	Gaining a healthy amount of weight is important for you and your baby	14,023 (1.82)	266	1.90	0.43	0	0	11

## Discussion

### Principal Findings

This observational study explored the use of a digital media campaign applying complementary tactics to increase awareness about healthy GWG among women in Alberta. Google AdWords targeted users who were actively searching for pregnancy-related information, while Facebook ads reached an audience who were not actively seeking this information. Both tactics directed users to an established website that had credible, evidence-based information, as well as helpful interactive tools to support women achieve a healthy GWG. Observational studies of digital campaigns and the sharing of lessons learned are important as health organizations continue to increase their presence on the Web and as Facebook increasingly limits the reach of unpaid messaging.

Although determining definitive benchmarks and comparing digital marketing tactics can be difficult because of the many variables involved (eg, budget, target audience, industry, competition, imagery, etc), the GWG digital campaign did exceed recently published standards. A digital marketing company in the United States investigated ad campaign metrics and determined that the average CTR across all industries in Google AdWords was 1.91% and average CPC was US $2.32 (Can $2.92) for paid search ads [28]. The average CTR for Facebook ads was 0.9% and average CPC was US $1.72 (Can $2.19) [29]. Using these overall benchmarks, our campaign had approximately 3 times the CTR for a third of the cost in Google AdWords and had approximately double the CTR for one-fifth of the cost in Facebook. The results of this campaign serve as the baseline for future campaigns and provide insights into target audience preferences and the components that contribute to successful advertising.

Previous health campaigns have used paid digital display ads (ads placed on various websites that are not affiliated with the advertiser) and search engine ads or have focused solely on social media platforms to reach target audiences. Cooper et al [30], for example, used Google ads to direct users searching for gynecologic cancer information to content on the Centers for Disease Control and Prevention website. Visits to the advertised webpages were 26 times higher during the 3-month campaign compared with the period before. Another campaign conducted in the state of Michigan over 11 weeks with a US $15,000 budget, used Facebook ads to increase awareness of a newborn screening and biobanking program [31]; the campaign reached 1.88 million users while achieving 15,958 website clicks, 452 shares, and 542 comments. Facebook ads have also been used to reach women for participation in prenatal research studies [22,32,33]. Compared with traditional tactics, Web-based ads were found to be more efficient and cost-effective for recruitment because of the ability to target women based on specific demographic and geographic features.

In this study, almost 80% of users who clicked on a Google ad completed a conversion on the website, which highlights the ability of these ads to attract highly interested users. Using Facebook ads instead of digital display ads as a complementary tactic also has several advantages, such as more effective targeting capabilities, higher-quality data on ad performance (which enabled us to refine the ad content between phases), ability to see user comments, the opportunity for 2-way communication, and extended organic reach when users interact with ads (because the engaged users’ social networks are seeing their engagement and thereby exposed to the ad message).

Online ads have been shown to impact users and increase awareness of the message even without a click [34]. A study involving a tobacco prevention campaign using Web-based ads to direct users to a campaign website found that exposure to digital display and search ads influenced visits to the website with added visits occurring up to 4 weeks after a user was exposed to an ad [35]. The ads were also found to influence other information-seeking behaviors, with users visiting additional cessation sites following ad exposure; this further emphasizes the benefit of complementary ad tactics as a user exposed to a social media ad may later use a search engine to research the topic, providing another opportunity to advertise and direct the user to a trusted, designated website.

A well-defined strategy including target audience research, aligning metrics with campaign goals, and a monitoring and evaluation plan was essential to the campaign. Lee et al [36] described a similar experience in using digital tactics as part of a campaign to increase awareness among health care professionals in California about smoking cessation resources for patients; the authors also comment on the benefits of having a clear purpose for each campaign tactic, continuous data monitoring to guide activities, and working with a marketing consultant. Hiring a vendor is an additional expense; however, organizations with limited experience may benefit from the technical expertise and the opportunity for staff to learn about campaign design and management. The GWG digital campaign began with the support of an external expert but advertising functions transitioned to internal staff as they grew more comfortable with the technology and system established by the external expert.

The strategy allowed for each phase of the campaign to build upon the data collected and lessons learned throughout the project. The ability to measure, monitor, and adapt the strategy continuously allowed for gradual optimization of campaign elements toward target audience preferences, lower costs, and a wider reach. Using readily available metrics, campaign activities were adjusted on the basis of components that indicated effectiveness. The highest-performing social media ad, for example, occurred in the last phase of the campaign and represented a combination of the most successful elements (eg, text, branding, and imagery) from previous ads. Furthermore, ad fatigue is a common challenge with digital advertising, where ad performance tends to decline over time. Running 2 Facebook ads at a time—and testing the 2 against one another—helped to reduce ad fatigue by keeping the content new and engaging while providing a means to test the performance of various campaign elements among the target audience.

### Limitations

This study has a number of limitations. Conversions could only be reported for Google AdWords because of an oversight in placing a tracking code in the HPHC website. Future digital campaigns will include this coding consideration early in the planning stage to ensure that additional data can be collected. There are also limitations in comparing results between marketing tactics as they reach different audiences and have different aims. Determining which tactic is a better value or which had a larger impact on the target audience is dependent on the individual interpretation that considers audiences and objectives—it would not be accurate in this case to directly compare the CTR for Google AdWords with the CTR for Facebook ads, for example.

This study did not assess the impact of the campaign on women’s behaviors, knowledge, or attitude; however, 83% of women in a study by Lagan et al [12] reported that the internet was used to influence decision making during their pregnancy. Women felt that their confidence levels to make decisions during pregnancy and their ability to engage in discussions with their health care provider significantly increased after using the internet. Thus, in the case of the GWG campaign, paid digital marketing may offer a means to meet women’s informational needs and ensure they receive credible, evidence-based, and personalized recommendations about GWG. Future research exploring the impact of digital ads on health-related attitudes and behavioral outcomes is warranted.

### Conclusions

This study adds to the literature by employing search engine marketing and social media advertising as complementary tactics for GWG health promotion goals. While the quality of information on the Web is highly variable, Google AdWords can be used by health organizations to promote credible websites as women search for specific information to fill knowledge gaps and inform decisions about their pregnancy. An advantage of incorporating Facebook ads into an overall digital marketing campaign is the ability to deliver information to women without their intervention, reaching those who may not be aware of health information related to GWG and women who may not have information-seeking skills.

Harnessing the power of search engine marketing and social media advertising together is a promising strategy to reach women who may not receive adequate information about GWG from other sources. Advantages of digital marketing include the opportunity to target a large audience and the ability to monitor and apply user data to optimize digital marketing activities. While the impact of digital marketing on behavior change is unknown, it may play an important role in contributing to increased awareness and knowledge of health topics that inspire action.

## Abbreviations

AHS: Alberta Health Services
CPC: cost per click
CTR: click-through rate
GWG: gestational weight gain
HPHC: Healthy Parents, Healthy Children
